# Validity and reliability of Arabic version of pediatric migraine disability assessment scale (Child Self-Report versus Parent Proxy-Report): a multi-center study

**DOI:** 10.1186/s10194-024-01713-6

**Published:** 2024-02-05

**Authors:** Rehab Magdy, Amr Hassan, Zeinab Mohammed, Mohamed A. Abdeltwab, Nawal F. Abdel Ghaffar, Mona Hussein

**Affiliations:** 1 Department of Neurology, Cairo University, Cairo, Egypt; 2 Department of Public Health and Community Medicine, Public Health and Community Medicine, Beni-Suef University, Beni-Suef, Egypt; 3 Department of Pediatrics, Beni-Suef University, Beni-Suef, Egypt; 4 Department of Neurology, Beni-Suef University, Salah Salem Street, Beni-Suef, 62511 Egypt

**Keywords:** PedMIDAS, Pediatric Quality of Life Inventory, Arabic-speaking pediatric patients, Monthly migraine days, Visual analogue scale

## Abstract

**Background:**

Pediatric Migraine Disability Assessment (PedMIDAS) is one of the most frequently used questionnaires to assess disability from migraine in pediatric patients. This work aimed to evaluate the validity and test–retest reliability of the Arabic version of the child self-report versus the parent proxy report PedMIDAS. We also aimed to test the agreement between children's and parents' reports of the scale.

**Methods:**

PedMIDAS was subjected to translation and back-translation, then applied to 112 pediatric patients fulfilling the migraine diagnostic criteria. This cross-sectional study was conducted on two visits, one week apart. At visit 1, the following data were obtained from the included pediatric patients: disease duration, migraine type, current treatment regimen, monthly migraine days (MMD) during the last month preceding the enrollment, and migraine intensity using the visual analogue scale. Then, each child and his parent were independently asked to fill out PedMIDAS and Child Self-Report of the Pediatric Quality of Life Inventory™ 4.0 (PedsQL™) to test the convergent validity of PedMIDAS. At visit 2, each child was requested to complete PedMIDAS again, and so was the parent to evaluate test–retest reliability.

**Results:**

Cronbach’s alpha was estimated to be 0.94 for each instrument. For the child-self report PedMIDAS, the average measure intraclass correlation coefficient (ICC) value was 0.992 (95%CI = 0.989–0.995), while it was estimated to be 0.990 for the parent-proxy report with 95%CI = 0.985–0.993, indicating excellent test–retest reliability for both instruments. The child-self report and the parent-proxy report PedMIDAS scores were significantly correlated with MMD, VAS, and all domains of the corresponding PedsQL, supporting convergent validity for both instruments. Agreement between parent and child on disability grading categories of PedMIDAS was substantial (κ = 0.644).

**Conclusion:**

The Arabic version of PedMIDAS was a valid and reliable instrument to assess disability from migraine in Arabic-speaking pediatric patients with migraine. Parent reports can be valuable as a complement to child reports for a comprehensive assessment of migraine.

**Supplementary Information:**

The online version contains supplementary material available at 10.1186/s10194-024-01713-6.

## Introduction

Primary headaches are a common health issue in the pediatric population. According to epidemiological data, tension-type headache is the most frequently reported type (17%), followed by migraines (11%); 8% are migraines without aura, and 3% are migraines with aura [1, 2]. The estimated prevalence of migraine among schoolchildren (6 to 18 years) in the Arab world ranged between 7.1% and 13.7% [3]. Migraine has been reported to have a substantial impact on child`s quality of life and psycho-social competence as it increases school absenteeism, decreases academic performance, and affects the child's interaction with his family and socialization with peers [4]. Pediatric migraine was also reported to be commonly associated with some psychiatric comorbidities such as depression, anxiety, adjustment, and conduct disorders [5, 6].

The Pediatric Migraine Disability Assessment (PedMIDAS) is one of the most frequently used questionnaires to assess how migraines impact the patient’s everyday life [7, 8]. The questionnaire was adapted by Hershey et al. [9] and validated for patients between the ages of 4 and 18. The tool structure consists of six questions about the impact of migraine on school absenteeism and leisure activities in the last three months. The questionnaire is based on another tool, i.e., the Migraine Disability Assessment (MIDAS), intended for adults aged 20–50, developed by Stewart et al. [10].

PedMIDAS was translated and validated into French [11], Brazilian Portuguese [12], and Italian [13]. However, the validity and reliability of the Arabic version have not yet been tested. It is worth mentioning that approximately 313 million people speak Arabic as a primary language [14].

As stated in the original validation study, PedMIDAS is used for a subjective assessment of migraine disability as perceived by a patient [9]. Although young children can reliably self-report health-related quality of life (HRQOL) using an age-appropriate instrument, a multi-informant approach, including concurrent reports from parents or teachers with child reports, is recommended to assess the child’s well-being comprehensively [15]. Furthermore, some situations potentially threaten the reliability of the child self-report when the child is too young, too cognitively impaired, too ill, or too fatigued [16]. So, a reliable and valid parent proxy report PedMIDAS is highly needed.

Hence, the main aim of the present study was to evaluate the validity and test–retest reliability of the Arabic version of child self-report versus parent proxy report PedMIDAS. We also aimed to test the agreement between children's and parents' reports of the scale.

## Methods

### Study design and eligibility criteria

This cross-sectional study was conducted on 112 children with migraine recruited from three headache clinics in two Arabic countries (Beni-Suef University Hospital, Kasr Al Ainy Hospital in Egypt, and Aseer Central Hospital in the Kingdom of Saudi Arabia,).

Patients aged 4–18 years who were literate in Arabic, who fulfilled the International Headache Society (IHS) criteria for migraine [17] with and without aura, episodic or chronic type, and who do not require significant adjustment to their treatment at study enrollment are included.

We excluded children who had received/changed prophylactic medications after completing the first questionnaire in the 1st visit, children with hemiplegic migraine, basilar-type migraine, retinal migraine, and complications of migraine (ICHD-II codes 1.2.4–1.2.6, 1.3–1.5) [17], medication overuse headache, concomitant chronic medical conditions. All patients had migraine as the main complaint at the time of assessment.

The study was conducted on two visits, approximately one week apart. At visit 1, each child was submitted to PedMIDAS and Child Self-Report of the Pediatric Quality of Life Inventory™ 4.0 (PedsQL™). Likewise, the parent was requested to complete PedMIDAS and Parent Proxy-Report of PedsQL™. After one week (visit 2), each child was requested to complete PedMIDAS again, and so was the parent to evaluate test–retest reliability.

Either father or mother was allowed to participate so that for every questionnaire the child filled out, there was a parallel one filled out by a parent. The instructor read the questionnaire for the child to ensure understanding of items and response ratings. On the other hand, the parents were requested to fill out the questionnaires independently in a room separate from the one where the child was interrogated unless the illiterate parents needed assistance with reading.

### Measures

1. Demographic data of both child and parent and headache characteristics were recorded, including disease duration, migraine type, and current treatment regimen.
2. Monthly migraine days (MMD) during the last month preceding the enrollment were extracted from the headache diary after ensuring compliance with writing down the number of headache days.
3. Migraine intensity using the visual analogue scale (VAS) [18].
4. The Pediatric Migraine Disability Assessment (PedMIDAS) [9]:

The English version of PedMIDAS is translated into Arabic “ forwards and backward” by the Languages and Translation Center, Cairo University (Supplementary material). The PedMIDAS consists of six questions concerning the impact of the ailment on school absenteeism, home life, and leisure activities. The degree is described as “low or none” in patients with a score of 10 or less. A mild degree is assigned to a patient with a score of 11–30 points, a “moderate” degree with a score of 31–50 points, and it is “severe” when the score is higher than 50 points [19]. The higher the score, the larger the negative impact on the patient’s quality of life.

5. The Pediatric Quality of Life Inventory™ 4.0 (PedsQL™) [20]

Two versions of the validated Arabic PedsQL were used: Child Self-Report and Parent Proxy-Report [21]. Children were asked to rate health problems they have experienced over the past month across four domains of HRQOL: physical domain (8 items) and 15 items for emotional, social, and school domains. Likewise, parents were asked to fill out the questionnaire to assess their perceptions of their children's HRQOL. Self-report and proxy-report items are identical but differ in first-person or third-person format.

Age-appropriate rating responses are used, as a 5-point Likert scale is utilized across child self-report for ages 8 – 18 (0 = never a problem; 1 = almost never a problem; 2 = sometimes a problem; 3 = often a problem; 4 = almost always a problem). In contrast, a 3-point Likert scale of emotional face icons is utilized in child self-report for ages 5–7 (0 = smiling face, 2 = neutral face, 4 = frowning face). Parent proxy-report response ratings follow the former scaling system (5-point response scale), regardless of the child’s age.

Items on the PedsQL Generic Core Scales are reverse-scored and transformed to a 0–100 scale (0 = 100, 1 = 75, 2 = 50, 3 = 25, and 4 = 0). Two subscale scores are generated: the psychosocial health summary score, which is the mean of the emotional, social, and school domains scores, and the physical health summary score, which is the same as the physical domain score. The total scale score is the mean of all items. Higher scores indicate a better HRQOL [20].

### Ethical statement

Written informed consent was obtained from the patients` parents, and verbal assent from the children. Data were confidential and anonymous. Ethical approval was obtained from the Research Ethics Committee, Faculty of Medicine, Beni-Suef University (approval number: FMBSUREC/03092023/Hussein).

### Statistical analysis

Data were analyzed using a statistical package for social sciences (SPSS) version 26. Numeric data was described using mean and standard deviation in the case of parametric data and the median and interquartile range with non-parametric data. For categorical data, frequencies and percentages were used. Internal consistency of the child self-report and parent-proxy report PedMIDAS scales were measured using Cronbach’s alpha for the total scale scores. In addition, Cronbach alpha if the item deleted was reported. Test–retest reliability for both scales scale was assessed using the intra-class correlation coefficient (ICC), the two-way mixed model [22], and the absolute agreement definition [23], which is more fit in assessing test–retest reliability, and single and average measures were reported. Values below 0.5 indicate poor reliability, between 0.5 and 0.75 indicate questionable reliability, 0.75 and 0.9 indicate good reliability, and any value above 0.9 indicates excellent reliability [23].

The Spearman's rho correlation coefficient was explored to assess the convergent validity for the child self-report and other scales used (VAS, MMD, and the total score of the Child self-report PedsQL™ scale and its domains), and for the Parent proxy report PedMIDAS and (VAS, MMD, and the total score of Parent proxy report PedsQL™ scale and its domains). A correlation coefficient of 0.1‐0.2 was considered poor, 0.3‐0.5 fair, 0.6‐0.7 moderate, and 0.8–0.9 very strong, and one perfect [24].

To test the agreement between the child self-report and parent-proxy report PedMIDAS raters, Cohen’s kappa coefficients (k) were calculated. Kappa ranged from -1 to = 1 where Kappa < 0 means Less than chance agreement, 0.01–0.20 Slight agreement, 0.21– 0.40 Fair agreement, 0.41–0.60 moderate agreement, 0.61–0.80 substantial agreement and 0.81–0.99 almost perfect agreement [25].

## Results

One hundred and twelve pairs of children (55 boys & 57 girls) and parents (22 fathers & 90 mothers) completed the questionnaires. The mean age of children was 12.36 ± 3.22, while the mean age of their parents was 42 ± 6. Detailed demographics of the included participants are illustrated in Table 1.

**Table 1:** Demographic data of the included children and their parents

Child demographics		
Age (mean ± SD)		12.36 ± 3.22
Age categories	< 12 y [n (%)]	40 (35.7%)
	≥ 12 y [n (%)]	72 (64.3%)
Gender	Male [n (%)]	55 (49.1%)
	Female [n (%)]	57 (50.9%)
Nationality	Egypt [n (%)]	86 (76.8%)
	Saudi Arabia [n (%)]	26 (23.2%)
Parents demographics		
Age (mean ± SD)		42 ± 6
Gender (n, %)	Male [n (%)]	22 (19.6%)
	Female [n (%)]	90 (80.4%)
Education (n, %)	Illiterate [n (%)]	8 (7.1%)
	Intermediate education [n (%)]	29 (25.9%)
	Highly educated [n (%)]	75 (67.0%)

*SD* standard deviation

Most of the children had episodic migraine (103, 92.0%), while only 9 cases had chronic type (8.0%). The median MMD experienced by our patients was 5 with IQR 4, while the median VAS score was 7 with IQR 2. Detailed headache characteristics of the included children are represented in Table 2.

**Table 2:** Migraine characteristics in the included patients

Migraine characteristics		Patients (*n* = 112)
MMD [median (IQR)]		5 (4)
VAS [median (IQR)]		7 (2)
Disease duration in years [median (IQR)]		2 (2)
Type of migraine	Episodic [n (%)]	103 (92.0%)
	Chronic [n (%)]	9 (8.0%)
Aura	Without Aura [n (%)]	81 (72.3%)
	With aura [n (%)]	31 (27.7%)
Prophylactic treatment	On treatment^a^ [n (%)]	83 (74.1%)
	No treatment [n (%)]	29 (25.9%)

*MMD* Monthly migraine days, *VAS* Visual analogue scale, *IQR* Interquartile range

^a^37 patients on propranolol, 34 on cyproheptadine, and 12 on valproate

At visit 1, the median score of the child-reported PedMIDAS was 44.5 with IQR 35, while that of the parent proxy report PedMIDAS was 47.5 with IQR 29.

### Reliability estimates of Arabic PedMIDAS

Child self-report and parent proxy report PedMIDAS showed excellent internal consistency, as Cronbach’s alpha was estimated to be 0.94 for each instrument (Table 3). For the child-self report PedMIDAS, the average measure ICC value was 0.992 (95%CI = 0.989–0.995), while it was estimated to be 0.990 for the parent-proxy report with 95%CI = 0.985–0.993, indicating excellent test–retest reliability for both instruments (Table 4).

**Table 3:** Reliability estimates by Cronbach's alpha for child self-report and parent- proxy report PedMIDAS

	Child self-report and PedMIDAS	parent- proxy report PedMIDAS
	0.94	0.94
	Cronbach's alpha if item deleted	
Q1	0.954	0.953
Q2	0.924	0.920
Q3	0.923	0.924
Q4	0.920	0.918
Q5	0.924	0.925
Q6	0.943	0.946

*PedMIDAS* Pediatric Migraine Disability Assessment

**Table 4:** Intraclass correlation coefficient (ICC) of for child self-report and parent- proxy report PedMIDAS (test re-test reliability)

**Child self-report PedMIDAS**							
	ICC	95% CI		F Test with True Value 0			
		Lower	Upper	Value	df1	df2	Sig
Single Measures	0.985	0.978	0.989	128.033	111	111	.000
Average Measures	0.992	0.989	0.995	128.033	111	111	.000
**Parent proxy report PedMIDAS**							
	ICC	95% CI		F Test with True Value 0			
		Lower	Upper	Value	df1	df2	Sig
Single Measures	0.980	0.971	0.986	99.400	111	111	.000
Average Measures	0.990	0.985	0.993	99.400	111	111	.000

*CI* confidence interval, *ICC* Intraclass correlation coefficient, *PedMIDAS* Pediatric Migraine Disability Assessment

### Convergent validity of Arabic PedMIDAS

The child-self report and the parent-proxy report PedMIDAS scores were significantly correlated with MMD, VAS, and all domains of the corresponding PedsQL, supporting convergent validity for both instruments.

The child-self report PedMIDAS scores showed a moderate correlation with MMD (*r* = 0.695), a fair correlation with scores of VAS (*r* = 0.560), child-self report PedsQL (*r* = -0.410) and its domains; physical health (*r* = -0.423) and the psychosocial health (*r* = -0.381) (Table 5).

**Table 5:** Convergent validity analysis of child self-report and parent proxy report PedMIDAS by Spearman correlation coefficients with the corresponding QoL variables and headache characteristics

		Child self-report PedMIDAS	
MMD		r	0.695
		p	0.000
VAS		r	0.560
		p	0.000
Child self-report PedsQL™	Physical health score	r	-0.423
		p	0.000
	Psychosocial health score	r	-0.381
		p	0.000
	Total score	r	-0.410-
		p	0.000
		Parent- proxy report PedMIDAS	
MMD		r	0.664
		p	0.000
VAS		r	0.571
		p	0.000
Parent proxy report PedsQL™	Physical health score	r	-0.425
		p	0.000
	Psychosocial health score	r	-0.356
		p	0.000
	Total score	r	-0.394
		p	0.000

*PedMIDAS* Pediatric Migraine Disability Assessment, *MMD* Monthly migraine days, *VAS* Visual analogue scale, *PedsQL™* The Pediatric Quality of Life Inventory™

The parent-proxy report PedMIDAS showed a moderate correlation with MMD (*r* = 0.664), a fair correlation with scores of VAS (*r* = 0.571), parent-proxy report PedsQL (*r* = -0.394), and its domains; physical health (*r* = -0.425) and the psychosocial health (*r* = -0.356) (Table 5).

### Agreement between self and parent reports

**Fig. 1:**
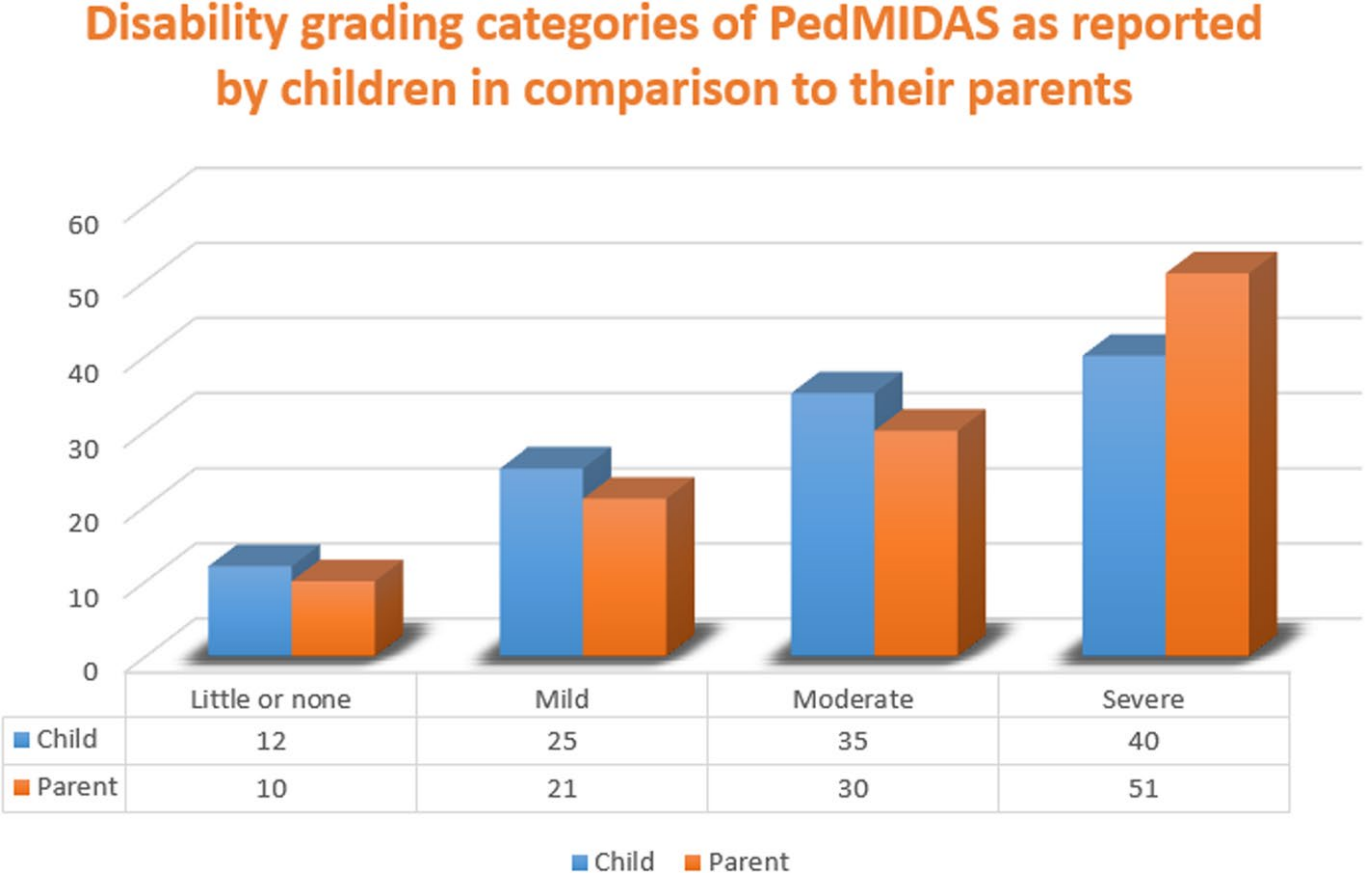
Disability grading categories of PedMIDAS as reported by children in comparison to their parents

The number of children and parents according to each of the disability grading categories of PedMIDAS is displayed in Fig. 1. It was noticed that the parents underestimated migraine disability reports than children's reports regarding some categories of PedMIDAS (little to none, mild, and moderate). On the other hand, the parents' reports overestimated the children's reports in severe disability grading (Fig. 1). Agreement between parent and child on disability grading categories of PedMIDAS was substantial (κ = 0.644) (Table 6).

**Table 6:** Agreement measure between child self-report and parent- proxy report PedMIDAS by Cohen’s kappa coefficients

Symmetric Measures				
	Kappa Value	Asymptotic Standard Error	Approximate T	Approximate Significance
Measure of Agreement	0.644	0.058	11.079	0.001

## Discussion

Parents are undoubtedly an informative provenience about their children's health and have input into decisions about care. Concurrent reports from parents with children's reports provide a convenient approach to assess the functioning of the child with migraine. Thus, while the validity and reliability of the child self-report PedMIDAS are satisfactory [9], the parent’s perspective is also relevant from a practical point of view.

In the current study, the Arabic version of PedMIDAS, either child-self report or parent proxy report, fitted the scaling assumptions, being a valid and reliable instrument to assess migraine disability in pediatric patients with migraine. The Arabic PedMIDAS had excellent internal consistency for either child-self report and parent proxy report as Cronbach’s alpha = 0.94, which was higher than the original PedMIDAS (α = 0.78), and other languages such as French (α = 0.76) [11], Brazilian Portuguese (α = 0.84) [12], and the Italian (α = 0.8) [13] versions.

Evidence exists that reduced physical activity, emotional problems and poor social functioning can all account for global disability in the pediatric population with migraines [26, 27], supporting the negative correlations observed in this study between scores of PedMIDAS and all domains of PedsQL. However, the magnitude of correlations between the scores of PedMIDAS and the different domains of PedsQL varied. A higher correlation was observed with physical than psychosocial health scores for child-self reports (*r* = -0.423 vs -0.381) and parent proxy reports (*r* = -0.425 vs-0.356). This could be explained by the target scope of the PedMIDAS-based questions, as they mainly focus on missed days or reduced productivity in schoolwork, household chores, and other leisure activities, all more relevant to physical rather than psychosocial functioning.

Although headache intensity is a major contributor to headache disability, more so than headache frequency [28], the correlations with MMD were moderate, while they were fair with VAS scores (*r* = 0.695 vs 0.560) and (*r* = 0.664 vs 0.571), for child-self reports and parent proxy reports PedMIDAS, respectively. A similar variation in the strength of correlations was inferred in the original validation work of PedMIDAS [9]. Children may report a higher intensity number on VAS than the real one, depending on the child’s view and his history of pain. In contrast, confusion is less likely with MMD by being compliant with a headache diary, in which a child is simply asked, “Did you have a headache today or not?” regardless of its severity [29].

This study is the first, to the best of our knowledge, to investigate agreement between parent proxy- and child self-reports of PedMIDAS in a sample of pediatric patients with migraine. In general, the perspectives of children and parents may vary across the health aspects investigated, as they agree more on the physical than the emotional or social aspects [30]. In PedMIDAS, the children were asked if their headache hindered them from being physically active at school or in their free time. This may explain the substantial agreement between children and their parents' reports described in this study (κ = 0.64).

Prior studies have acknowledged the agreement of child self-report and parent proxy reports on headache disorders. In line with the current results, Kröner-Herwig et al. [31] described a moderate parent–child agreement regarding headache frequency in a large pediatric sample aged 7–14. However, our results contrast studies that examined the parent–child agreement among older age groups in assessing the pain reports of headaches. In a Swedish study [32], a sample of students in 8th grade was interviewed, asking about the frequency of their headaches that their parents poorly agreed with. Another study found relatively low agreement between parents and adolescents (aged 13–18 years) on the presence or absence of headaches (κ = 0.39) [33].

Another point worth mentioning is that the parent proxy report PedMIDAS should not be considered a substitute for the child self-report PedMIDAS. We believe that each perspective represents a distinctive subjective reality, and both are important to be involved in clinical and research encounters. Hence, Smith et al. [34] emphasized that the reliability of parent proxy report may be affected by the projection of the parent’s feelings, beliefs and assumptions.

Some limitations might lessen the generalizability of the current results, such as the small number of patients, the small mean monthly migraine days, and the presence of 74% of the patients on prophylactic treatment. Also, the study was conducted during the school year, so responses may differ from the summer holiday based on findings from Heyer et al. [35]. Additionally, some parental factors may influence the parent–child agreement, including gender and educational level. The agreement levels are generally much higher when the raters are highly educated and are mothers rather than fathers [30]. Only 19.6% of our sample were fathers, and only 7.1% were illiterate, which may account for our satisfactory agreement results.

## Conclusion

The Arabic version of PedMIDAS, either child-self report or parent proxy report, is a valid and reliable instrument to assess migraine disability in Arabic-speaking pediatric patients with migraine. Consequently, parent reports can be valuable as a complement to child reports for a comprehensive assessment. Given the moderate agreement between parent proxy and child self-report of PedMIDAS, it may be reasonable to substitute parent proxy report for child self-report only when the child is too sick or unavailable.

### Supplementary Information

**Additional file 1.**

## Data Availability

Authors report that the datasets used and/or analyzed during the current study are available from the corresponding author upon reasonable request.
