# Supplementary material for: Validity and reliability of Arabic version of pediatric migraine disability assessment scale (Child Self-Report versus Parent Proxy-Report): a multi-center study
Source: J Headache Pain. 2024 Feb 5;25(1):15. doi: 10.1186/s10194-024-01713-6 (PMC10840291; doi:10.1186/s10194-024-01713-6)
Supplement: Supplementary file 1 — Additional file 1. [file 10194_2024_1713_MOESM1_ESM.docx]

**PedMIDASالنسخه العربية لاستبيان**

**يجب أن تعتمد إجاباتك على آخر 3 أشهر، حاول تقديم أفضل تخمين لديك**

|  | كم عدد أیام الدراسة التي فاتتك بالكامل في آخر 3 أشھر بسبب الصداع؟ |  |
| --- | --- | --- |
|  | كم عدد أیام الدراسة التي فاتتك بشكل جزئي في آخر 3 أشھر بسبب الصداع (لا تشمل الأیام الكاملة المحسوبة في السؤال الأول)؟ |  |
|  | كم یوما في الأشھر الثلاثة الماضیة عملت أقل من نصف قدرتك في المدرسة بسبب الصداع (لا تشمل بما في ذلك الأیام المحسوبة في السؤالین الأولین)؟ |  |
|  | كم عدد الأیام التي لم تتمكن فیھا من القیام ببعض الأعمال في المنزل (مثل الأعمال المنزلیة ، الواجبات المدرسيه ، إلخ) بسبب الصداع؟ |  |
|  | كم عدد الأیام التي لم تشارك فیھا في أنشطة أخرى بسبب الصداع (مثل اللعب ، الخروج ، الریاضة ، إلخ)؟ |  |
|  | كم یوما شاركت في ھذه الأنشطة ، لكن تعمل بأقل من نصف قدرتك (لا تشمل الأیام المحسوبة في السؤال الخامس)؟ |  |
| مجموع الدرجات | |  |
